# Lithium in drinking water and suicide risk

**DOI:** 10.17179/excli2022-4689

**Published:** 2022-03-03

**Authors:** Tomoyuki Kawada

**Affiliations:** 1 Department of Hygiene and Public Health, Nippon Medical School

## ⁯

Recent reports are indicating an inverse association between lithium in drinking water and suicide mortality (Liaugaudaite et al., 2022[6]; Izsak et al., 2022[3]). These reports basically adopted environmental monitoring data of lithium exposure and cause-specific mortality risk for a population. Caution should be paid to ecological data, from which individual suicide risk cannot be directly determined.

A meta-analysis determined that there was an inverse relationship between lithium in drinking water and suicide rates (Eyre-Watt et al., 2021[2]). To evaluate the relationship of lithium in drinking water with suicide rates, they reported correlation coefficients with 95 % confidence intervals (CIs). However, almost all of the component papers reported the beta value from simple and/or multiple regression analysis. I believe that the odds ratio (OR) or hazard ratio should also be adopted as an indicator of the effect size. They also observed differences in the association according to sex as there was an inverse relationship observed for only in men. Barjasteh-Askari et al. (2020[1]) conducted a meta-analysis, and the pooled ORs (95 % CIs) of the lithium concentration in drinking water for suicide mortality in men was 0.54 (0.35–0.84) and that in women was 0.70 (0.48–1.01). Recently, Izsak et al. (2022[3]) observed a significant relationship between these two variables only in men. Although the sex difference in the inverse relationship may not be easily explained, biopsychosocial factors contribute to these differences in the relationship.

The inverse ecological association should be verified by individual data on the biological monitoring of lithium exposure, such as between the serum concentration of lithium and the number of suicide events. Case-control studies analyzing this have been reported. Kanehisa et al. (2017[4]) reported that the adjusted OR (95 % CI) of subjects with a suicide attempt for a low serum lithium level was 0.228 (0.059–0.883). They considered that higher serum lithium levels might be protective against suicide attempts in lithium therapy-naïve individuals. Kurosawa et al. (2018[5]) reported that the adjusted OR (95 % CI) of the log-transformed lithium level for a suicide attempt was 0.156 (0.038–0.644). Prospective/intervention studies are needed to verify the causal inverse relationship of these variables and to speculate the mechanism for this relationship.

Finally, Szklarska and Rzymski (2019[8]) reported a review on the biological action, bioavailability, and metabolism of lithium, with special emphasis on the lithium content, as a fortifying food for the primary prevention of mood disorders and suicide attempts. They cited a paper by Schrauzer (2002[7]), reporting that the provisional recommended daily allowance of lithium for a 70 kg adult is 1,000 µg. The benefits gained from the appropriate intake of lithium as a useful micronutrient should be validated in further studies. 

## Declaration

### Conflict of interest

The author declares no conflict of interest.

### Financial disclosures

None.

## References

[1] Barjasteh-Askari F, Davoudi M, Amini H, Ghorbani M, Yaseri M, Yunesian M (2020). Relationship between suicide mortality and lithium in drinking water: A systematic review and meta-analysis. J Affect Disord.

[2] Eyre-Watt B, Mahendran E, Suetani S, Firth J, Kisely S, Siskind D (2021). The association between lithium in drinking water and neuropsychiatric outcomes: A systematic review and meta-analysis from across 2678 regions containing 113 million people. Aust N Z J Psychiatry.

[3] Izsak B, Hidvegi A, Balint L, Malnasi T, Vargha M, Pandics T (2022). Investigation of the association between lithium levels in drinking water and suicide mortality in Hungary. J Affect Disord.

[4] Kanehisa M, Terao T, Shiotsuki I, Kurosawa K, Takenaka R, Sakamoto T (2017). Serum lithium levels and suicide attempts: a case-controlled comparison in lithium therapy-naive individuals. Psychopharmacology (Berl).

[5] Kurosawa K, Terao T, Kanehisa M, Shiotsuki I, Ishii N, Takenaka R (2018). Naturally absorbed polyunsaturated fatty acids, lithium, and suicide-related behaviors: A case-controlled study. J Affect Disord.

[6] Liaugaudaite V, Raskauskiene N, Naginiene R, Mickuviene N, Sher L (2022). Association between lithium levels in drinking water and suicide rates: Role of affective disorders. J Affect Disord.

[7] Schrauzer GN (2002). Lithium: occurrence, dietary intakes, nutritional essentiality. J Am Coll Nutr.

[8] Szklarska D, Rzymski P (2019). Is lithium a micronutrient? From biological activity and epidemiological observation to food fortification. Biol Trace Elem Res.

